# First Description of the Adult Male of the Gall-Like Scale Insect *Allokermes galliformis* (Riley) (Hemiptera: Coccomorpha: Kermesidae)

**DOI:** 10.3390/insects10080250

**Published:** 2019-08-14

**Authors:** Kyle D. Krutil, Alison L. Hall, Whitney S. Cranshaw, Boris C. Kondratieff, Rachael A. Sitz

**Affiliations:** 1 Department of Bioagricultural Sciences and Pest Management, Colorado State University, Fort Collins, CO 80523-1177, USA; 2 USDA Forest Service, Rocky Mountain Research Station, 1221 South Main Street, Moscow, ID 83843, USA

**Keywords:** kermes scale insect, pin oak kermes, *Quercus* sp., drippy blight disease, scanning electron microscopy

## Abstract

The adult male of *Allokermes galliformis* (Riley, 1881) (Hemiptera: Coccomorpha: Kermesidae) is described for the first time in Colorado, United States of America. This scale insect species recently emerged as a significant pest of red oaks in Colorado through its causative role in drippy blight disease. A description and illustration of the adult male characterize its key external morphological characteristics.

## 1. Introduction

Kermes scales (Hemiptera: Kermesidae) are an understudied group of insects that feed on the sap of trees and shrubs in the family Fagaceae throughout the Nearctic, Oriental, and Palearctic regions [1,2]. In total, there are 92 species within Kermesidae [3], and in the Nearctic region there are 34 species representing five genera (*Allokermes*, *Eriokermes*, *Kermes*, *Nanokermes*, and *Olliffiella*) [4]. Kermes scales display marked sexual dimorphism [5]. Females have three nymphal instars and a pre-reproductive and post-reproductive adult stage, both of which have a gall-like form. Males typically exhibit four nymphal instars, with the “prepupal” and “pupal” instars occurring in a white cocoon-like test, and a winged adult stage [2,5].

Immature stages and the adult male kermes scales are minute in size and spatially separated throughout the landscape, which makes them difficult to collect. Furthermore, adult males are short-lived, lacking functional mouthparts. The sclerotized post-reproductive female is the most conspicuous and most readily collected life stage because they remain on branches long after oviposition. Kermes scale identification is largely based on morphological characteristics of pre-reproductive females, which can only be collected during a narrow phenological window, and their waxy covering may change throughout development [6]. Characterizing the morphology of the adult male life stage may help support the current phylogenetic placement of Kermesidae, which is based on morphological characteristics [7]. While host and locality information is available on each of the 12 species within the genus *Allokermes,* the specimens presented may also help disentangle the phylogenetic placement of species within *Allokermes* as well as aid in species identifications. A detailed description of the adult male is most needed for the type species of the genus *Allokermes*, the pin oak kermes *Allokermes galliformis* (Riley, 1881) [8]. Not only is *A. galliformis* one of the most widespread and well-studied species in the genus [1,4,9,10,11], it was also recently reported in outbreak numbers in Colorado, United States of America, where it was documented damaging red oak trees (*Quercus* spp.) in conjunction with the bacterial pathogen involved in drippy blight disease (*Lonsdalea quercina*) [12,13]. In these drippy blight diseased systems, *A. galliformis* exhibited a univoltine life cycle similar to the previously reported life cycle of *A. kingii* Cockerell, 1898 [14,15]. Furthermore, adult males typically congregated on large branches and trunks of host trees and surrounding debris [11].

*Allokermes galliformis* was originally described from female syntypes from Iron Mountain, (St. Francois County) Missouri [4]. Morphological descriptions are available for all female life stages [9,10]. Although the second-instar male was described [9], the adult male has not been described. Information on the adult male stage for species of the genus *Allokermes* is generally lacking with only the male of *A. kingii* (Cockerell, 1898) described [15]. The abundance of adult male scales on drippy blight diseased trees in northeastern Boulder County, Colorado, on planted northern red oak (*Quercus rubra* L.) has allowed us to provide a detailed morphological description of the adult male of *A. galliformis.*

## 2. Materials and Methods

### 2.1. Materials

The identification of *Allokemes galliformis* females feeding on red oak trees in Boulder, Colorado, USA, was determined by the morphological characteristics of pre-reproductive adults [11]. *Allokermes gillettei* (Cockerell) can also be found in the region, but this species is host specific to gambel oak (*Q. gambelii*) [10]. In this study, adult males were field-collected in July 2015 in Boulder, Colorado, USA (Colorado, Boulder, 40°01′72.89″ N, -105°25′99.04″ W, 2.vii.2015, R. Sitz). Adult males were aspirated from the trunk of infested northern red oak trees or reared in the lab from tests. The phenology of the males in relation to *A. galliformis* females, coupled with the red oak host plant association, led to the male species determination of *Allokermes galliformis*.

Specimens were stored in 90% ethanol at 4 °C until slide mounting or scanning electron microscopy. All slide mounted specimens and additional voucher specimens preserved in 80% ethanol were deposited in the C.P. Gillette Museum of Arthropod Diversity, Colorado State University, Fort Collins, Colorado. Additional specimens are stored in the Department of Bioagricultural Sciences at Colorado State University in 95% ethanol at −20 °C, as they may be useful in future molecule-assisted species identifications.

### 2.2. Slide Mounting

Males were cleared in a 5% KOH solution for 7–8 h [6], and then mounted on slides in Hoyer’s mounting medium. The slides were left in an oven at 46 °C for 7–10 days, then the edges of the coverslips were sealed using clear nail polish. Measurements were conducted with an Olympus BHA Phase System compound microscope following previous descriptions [2,15,16,17,18]. Measurements are in micrometers (μm), and 6 specimens (n = 6) were used for each measurement. Ranges are reported, followed by the mean in parentheses. Table 1 presents the abbreviations used for structures, and abbreviations follow previous literature [1,15,16]. Accurate measurements of the radius (rad) and media (med) of the wing could not be taken due to folding of the wings in slide-mounted specimens.

### 2.3. Scanning Electron Microscopy

For scanning electron microscopy, specimens were initially prepared by fixing in 2.5% glutaraldehyde in 0.15 M sodium phosphate buffer, pH 7.4, at 4 °C for 24–48 h. Post-fixation was carried out in 1% buffered osmium tetroxide overnight at 4 °C, then samples were dehydrated through a graded ethanol series, followed by critical point drying using a BioRad E3000 critical point dryer (Quorum Technologies, East Sussex, England), or by dehydration with acetone and hexamethyldisilazane. All samples prepared for SEM were sputter coated with 10 nm gold using a Hummer VII (Anatech USA, Union City, California, USA). Micrographs were taken using a JEOL JSM-6500F Field Emission Scanning Electron Microscope at the Central Instrument Facility, Imaging Laboratory, located at Colorado State University. All images were captured digitally as tiff files, and graphically edited to input black image backgrounds (Adobe Photoshop, CS6 13.0.1). All measurements were taken at the widest or longest point of a given structure (Table 1), and are given in μm. Ranges are presented, followed by the means in parentheses.

## 3. Results

**Figure 1:**
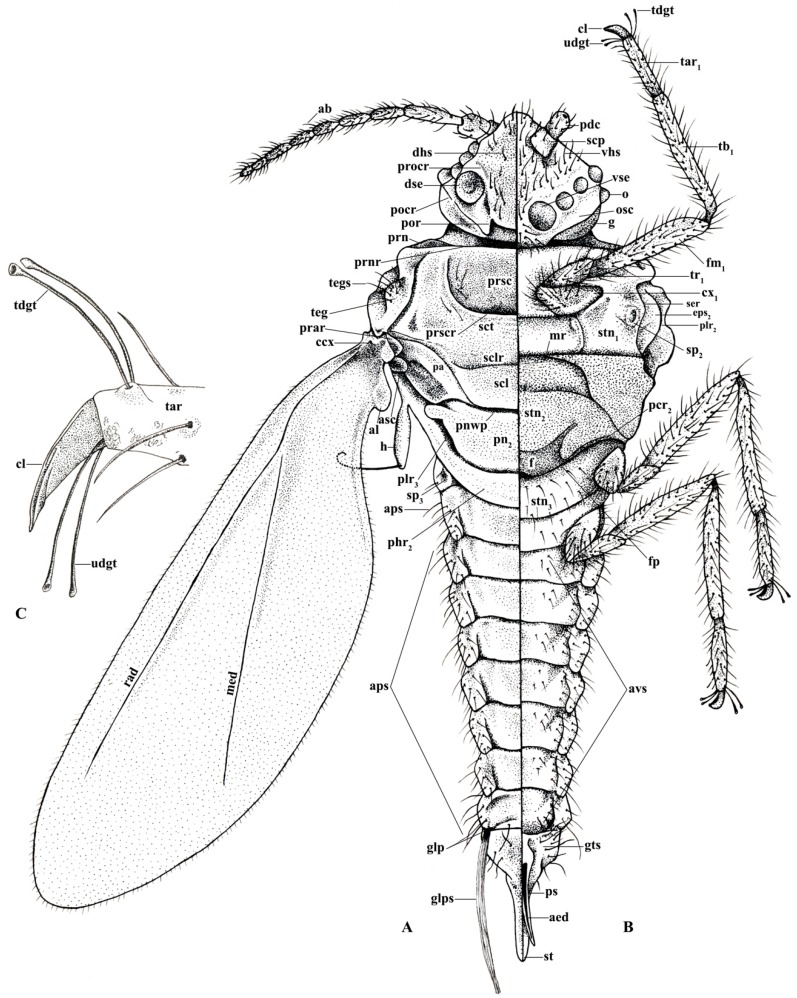
Illustration: (**A**) Dorsal view of *Allokermes galliformis*; (**B**) Ventral view of *A. galliformis*; (**C**) Claw, tarsus, and ungual digitules. Illustration by Alison L. Hall.

**Figure 2:**
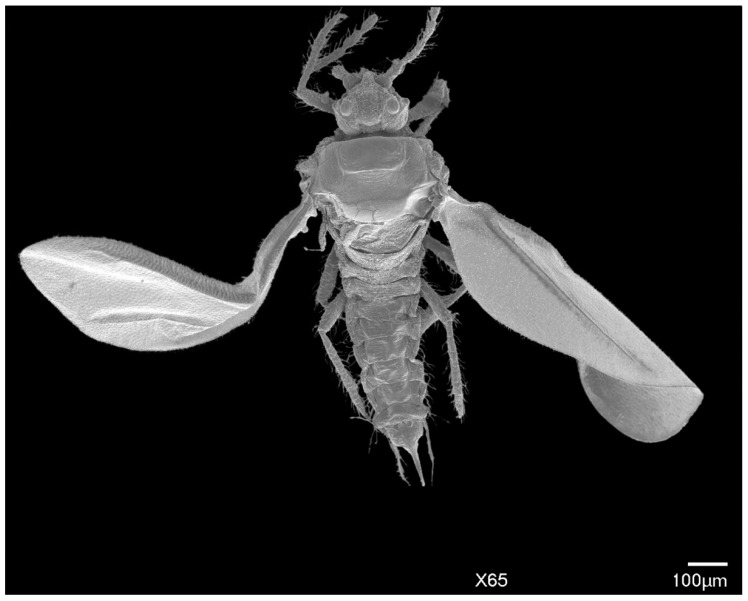
SEM dorsal view of the entire male *Allokermes galliformis*.

In life, adult males are orange to brown with clear to iridescent wings. Excluding the head, the body shape is robust, subtriangular with long, distinct legs, and an elongated genital segment (Figure 1A,B, Figure 2). Many specimens observed were missing sections of antennae, legs, or wings. Fusion of consecutive antennal segments was observed in some specimens, but all antennal segment measurements were taken from antennae without fusion. Folding of the wings in mounted specimens did not permit accurate wing and wing vein measurements. Total body length from the apex of the head to the apical tip of the penile sheath is 976–1091 μm (mean 1022 μm); the width at the mesothorax is 277–395 μm (mean 325 μm) at the scutum.

### 3.1. Description of the Adult Male of Allokermes galliformis (Riley, 1881)

#### 3.1.1. Head

**Figure 3:**
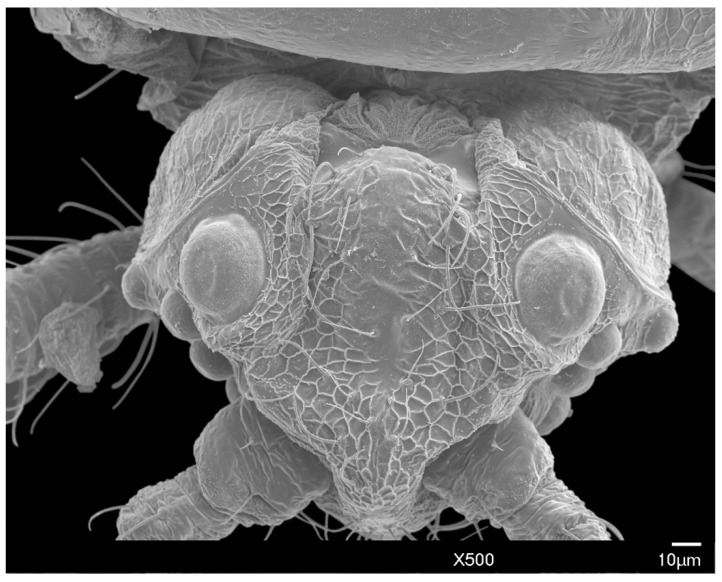
SEM dorsal view of the male *A. galliformis* head and raised net-like cuticular patterning, showing the dorsal simple eyes, post occipital ridge, post ocular ridge, scape, and genae.

**Figure 4:**
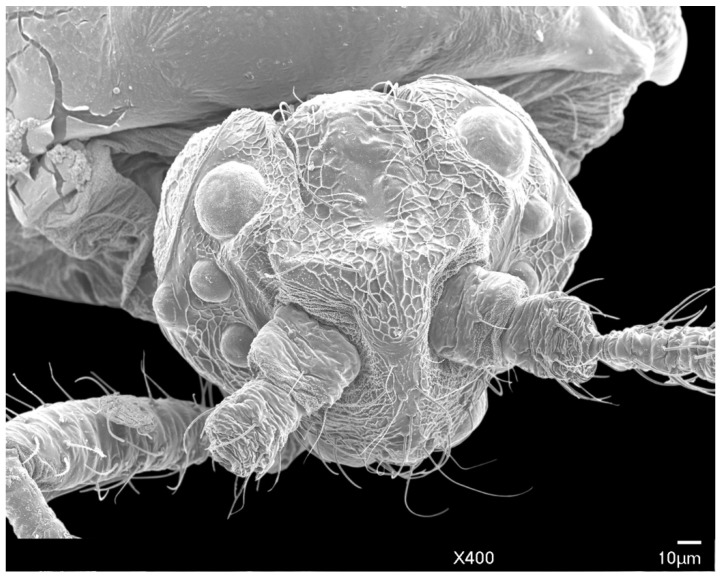
SEM dorso-anterior view of the male *A. galliformis* head, showing the post occipital ridge, post ocular ridge, ventral simple eyes, ocelli, scape, pedicel, ventral simple eyes, and genae.

**Figure 5:**
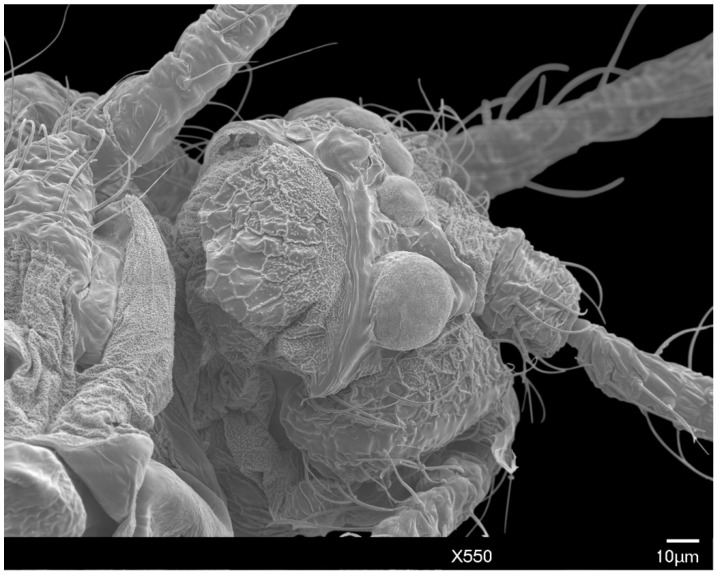
SEM lateral view of the male *A. galliformis* head showing the ventral simple eyes, ocellus, pedicel, and genae.

**Figure 6:**
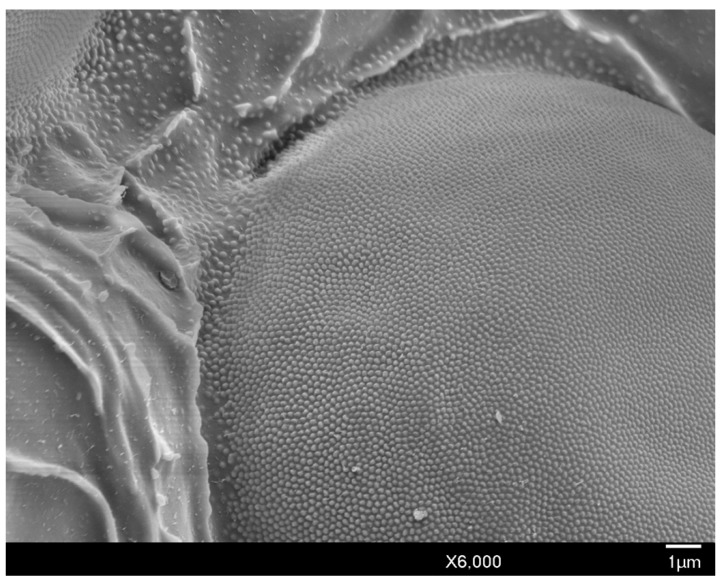
SEM close-up of the granular surface of the head and dorsal simple eye of male *A. galliformis*.

The head is relatively angular, and distinctly separate from the thorax. Dorsally, the head is approximately pentagonal with rounded edges, and is generally ovoid laterally (Figure 3 and Figure 4). The head length from the apex to pronotal ridge (prnr) is 109–143 μm (mean 123 μm), and the width across genae (g) is 162–208 μm (mean 179 μm). Each ocular sclerite (osc) containing a single row of 5 simple eyes [19] wrapped laterally around the head, and ocelli (o) present distal to the row of simple eyes (Figure 5). Ocular sclerites are not reticulated. Dorsal and ventral simple eyes each have a granular surface (Figure 6). The diameter of the dorsal simple eye (dse) is 29–40 μm (mean 34 μm); the diameters of ventral simple eyes (vse) from the most proximal to most distal point are 19–22 μm (mean 21 μm), 15–19 μm (mean 16 μm), 17–20 μm (mean 18 μm), and 32–38 μm (mean 34 μm), respectively; the diameter of ocelli is 15–19 μm (mean 16 μm). The cuticle of head exhibits polygonal reticulation; setae present dorsally and ventrally. Dorsal head setae (dhs) are comparatively sparse and approximately central but not on the mid-cranial ridge; ventral head setae (vhs) are more numerous over much of the ventral surface (Figure 3). Dorsally, the postoccipital ridge (por) is well-developed, sclerotized, approximately M-shaped, converging with the postocular ridge (pocr) and preocular ridge (procr).

*Antennae:* The filiform, has 10 segments, with distinct scape (scp) and pedicel (pdc); the total antennal length is 500–584 μm (mean 529 μm); the mean ratio of body length to antennal length is 1:1.94 μm. The antennal segments all bear numerous long setae. The scape is 32–55 μm (mean 41 μm) in length, widest at the base 38–44 μm (mean 41 μm), with few setae. The pedicel is 46–53 μm (mean 49 μm) in length, 32–32 μm (mean 32 μm) in width at the base, with numerous setae. Table 2 gives the mean length and width of antennal segments.

#### 3.1.2. Body Setae and Pores

**Figure 7:**
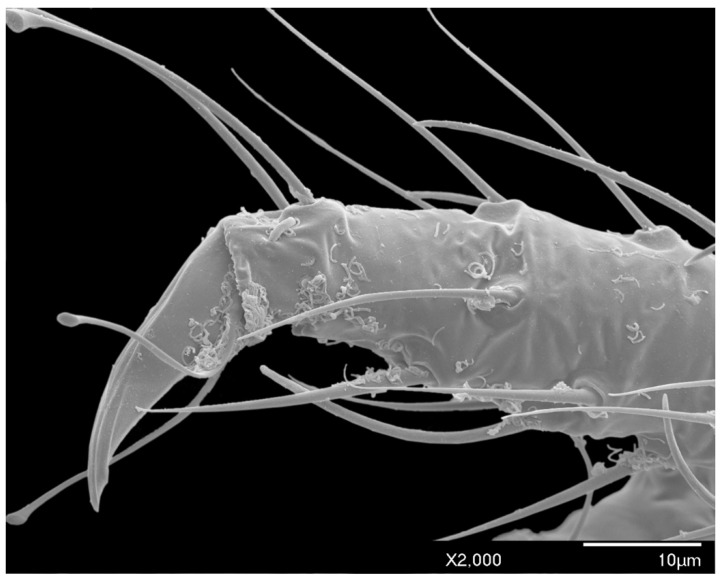
SEM of the male *A. galliformis* tarsus, tarsal digitules, ungual digitules, and claw. Wax excretions are present on the tarsus and proximal portion of the claw.

**Figure 8:**
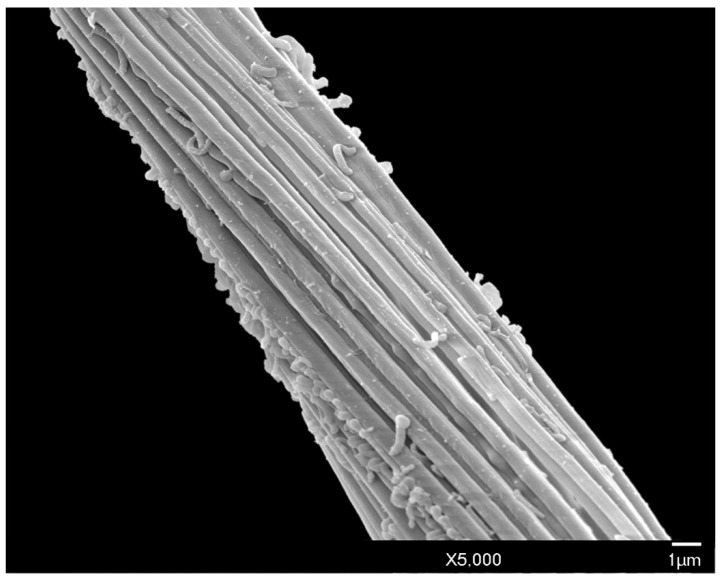
SEM close-up of male *A. galliformis* showing the setae of the glandular pouch composed of wax filaments.

**Figure 9:**
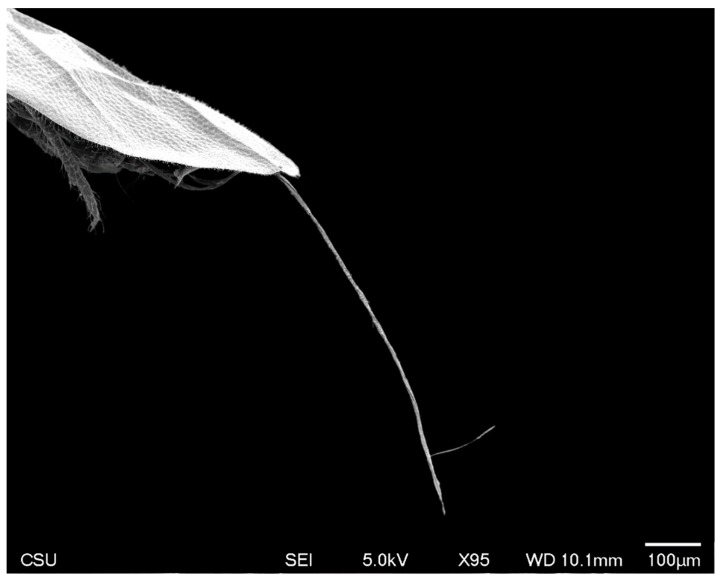
SEM showing the full length of one seta of the glandular pouch of the male *A. galliformis*.

Long, conspicuous setae are present on most of the head, body, and appendages. Much of the exoskeleton has a granular surface, including the simple eyes (Figure 6). Polygonal reticulation is present on the head (Figure 3, Figure 4 and Figure 5). Waxy excretions are especially prominent on the genital segment, tarsus (tar) near the claw (cl) (Figure 7), and on and around the glandular pouch (glp) and glandular pouch setae (glps) (Figure 8). The genital segment also bears setae (gts). Glandular pouch setae (glps)are made of wax filaments (Figure 8 and Figure 9).

#### 3.1.3. Thorax

The thorax measures 357–424 μm (mean 395 μm) in length, and 378–433 μm (mean 416 μm) in width.

*Prothorax:* Small lateral pronotal sclerite (prn) are well-defined, approximately triangular; the pronotal sclerite and pronotal ridge are situated posterior to the head. The ventral prosternum (stn_1_) is 172–202 μm (mean 183 μm) in length and 332–407 μm (mean 370 μm) in width, articulating anteriorly with the posterior edge of procoxa (cx_1_); the posterior edge of the prosternum is situated anterior to the marginal ridge (mr) of the basisternum.

**Figure 10:**
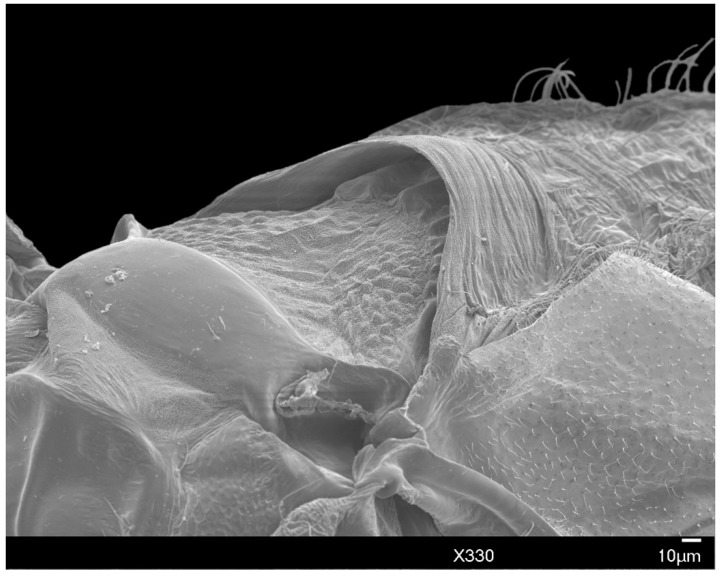
SEM prescutum and raised prescutal ridge of male *A. galliformis*.

*Mesothorax:* Widest point of the body. The prescutal ridge (prscr) is 63–84 μm (mean 75 μm) in length and 126–139 μm (mean 132 μm) in width, laterally raised over the less-sclerotized prescutum, similar to an “awning” (Figure 10). The dorsal alinotum comprises the prescutum (prsc), scutum (sct), and scutellum (scl). The prescutum subrectangular, comparatively dorsoventrally compressed, is situated beneath the raised prescutal ridge (Figure 10). The scutum is strongly sclerotized, 164–218 μm (mean 197 μm) in length and 277–395 μm (mean 325 μm) in width, lying posterior to the prescutal ridge. The scutellum is situated posterior to the scutum, sub-rhomboid-shaped, 59–67 μm (mean 64 μm) in length and 189–218 μm (mean 200 μm) in width. The scutum is separated from the scutellum by the scutellar ridge (sclr). The mesopostnotum (pn_2_), situated posterior to the scutellum, is posteriorly rounded with a distinct anterio-distal curve. The scutellum and mesopostnotum are separated by the posterior notal wing process (pnwp); the metapleural ridge (plr_3_) runs along the posterior margin of the mesopostnotum. Mesopostphragma (phr_2_) are weakly sclerotized, situated posterior to the axillary cord and articulating with sublateral hamulohalteres on each side.

The dorsolateral portion of the mesothorax has a prealare ridge (prar). Tegula (teg) have 6–8 tegular setae (tegs). A small, rounded additional sclerite (asc) is present on the mesothorax and posterior to the hamulohaltera (h). Prealare and this additional sclerite articulate sublaterally with the costal complex of the wing vein (ccx).

Laterally, the mesepisternum (eps_2_) is proximal to the subepisternal ridge (ser). Postalare (pa) is situated dorsal and posterior to the mesepisternum. The basisternum (stn_2_) is a large, subrectangular, shield-like plate with margins strongly sclerotized, 105–143 μm (mean 128 μm) in length and 202–231 μm (mean 214 μm) in width at the widest point. The anterior edge of the basisternum is bordered by the marginal ridge; the posterior edge is bordered by the precoxal ridge (pcr_2_), and partially covers the furca (f) and furcal pit (fp).

**Figure 11:**
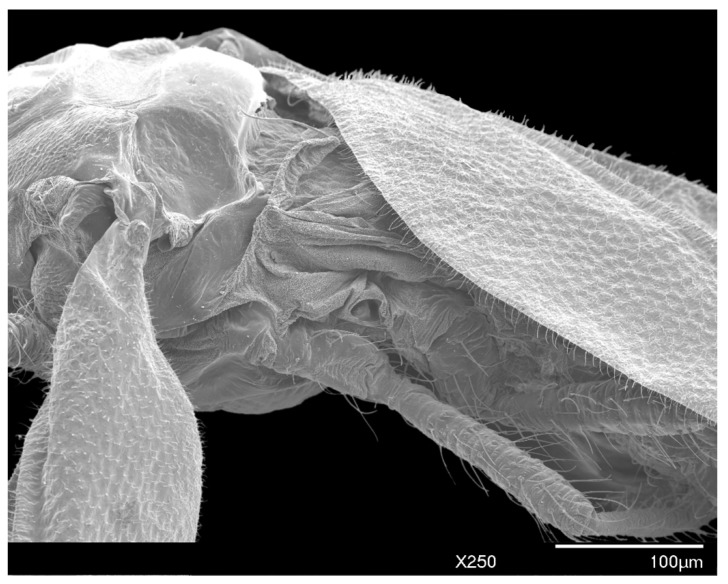
SEM lateral view of the peritreme of the metathoracic spiracle metaepisternum of male *A. galliformis* also showing the microtrichia, costal complex of the wing, alar, and additional sclerite.

*Spiracles:* The mesothoracic spiracle (sp_2_) and metathoracic spiracle (sp_3_) are situated sublaterally, with peritreme widths measuring 12–19 μm (mean 14 μm) and 12–14 μm (mean 13 μm), respectively, at the widest point. The peritreme is large and distinct (Figure 11).

*Metathorax:* Indistinct and weakly sclerotized. The metasternal plate is not visible. There are 11–19 (mean 16) ventral setae. Dorsal setae are absent.

*Wings:* Each wing is 820–943 μm (mean 868 μm) in length, extending past the apex of the abdomen and with a distinct alar lobe (al) (Figure 1A). Small, numerous microtrichia cover all wing surfaces and surround the wing margins. Media arise along the radius at approximately 1/4 the total length of the radius, distal to the costal complex of the wing veins. The radius extends approximately 4/5 the entire length of wing, with a slight proximal curve in the first 1/4, before conjunction with the media. Hamulohalteres are finger-like, measuring 78–86 μm (mean 82 μm) in length and 17–23 μm (mean 21 μm) in width, with an apically hooked hamulus seta arising at the distal apex, approximately equal in length to the hamulohaltere. The mean ratio of total body length to wing length is 1:1.18 μm.

*Legs:* Pro- and mesothoracic legs are almost subequal in length, with the metathoracic legs being shortest. The mean ratio of prothoracic leg to total body length is 1:0.58 μm. The mean claw length is about 22 μm, similar to the width of the tarsal base. The tarsus has two tarsal digitules (tdgt), each 25–32 μm (mean 29 μm) in length, arising from the dorsal to the claw. The claw has two ungual digitules (ugdt), each approximately the same in length as the claw, arising ventrally at the point of attachment of the claw to the tarsus (Figure 7). Both tarsal and ungual digitules are knobbed apically (Figure 1C and Figure 7). All leg segments, except for the claw, are covered with numerous setae (Figure 1A and Figure 2). Table 3 provides the mean length and width of each leg segment.

#### 3.1.4. Abdomen

The abdomen consists of eight pregenital segments; the length, including the apex with the genital segment is 512–655 μm (mean 547 μm); the width at the third abdominal segment is 202–256 μm (mean 231 μm). All abdominal segments have numerous long setae (aps). There are 9–18 (mean 13) abdominal ventral setae (avs) on each of segments 2–4; 5–15 (mean 10) on each of segments 5–7; 1–3 (mean 2) on abdominal sternite 8, and 6–15 (mean 11) on the genital segment. Dorsal setae are absent. Setae of the glandular pouch (glps) are present in two bundles; each seta is about 123 μm in length, arising from the glandular pouch on abdominal segment 8, often together with numerous wax excretions along the entire length (Figure 8 and Figure 9).

*Genital segment:* The style (st), is 101–122 μm (mean 113 μm) in length from the rounded apex to the posterior margin of abdominal segment 8, extending beyond the penial sheath (ps). The penial sheath, from the apex to the basal ridge, is 101–122 μm (mean 113 μm) in length and 23–25 μm (mean 25 μm) in width at the widest point. The aedeagus (aed), with distinctly sclerotized margins, lies within the penial sheath. In lateral view, the aedeagus curves slightly ventrally away from the style. Approximately six genital segment setae (gts) are present on each side of the ninth abdominal sternite. Excretions of wax are numerous on and around the style and genital segment.

## 4. Discussion

Prior to this work, the only description of adult male *Allokermes* available was that of *A*. *kingii*, collected at Blacksburg, (Montgomery County) Virginia [15]. A comparison of this male with that of *A. galliformis* is now possible, and we found several diagnostic characteristics unique to *A. galliformis* as compared to *A. kingii*. In *A. galliformis,* the head is approximately subtriangular (it is rounded in *A. kingii*); the body is substantially more robust at the scutum and more tapered posteriorly in *A. galliformis* than in *A. kingii*; furthermore, the genital segment is more abruptly narrowed in *A. galliformis* than in *A. kingii*. *Allokermes galliformis* also lacks the dorsal abdominal setae that are present in *A. kingii*. 

When compared to males of species in a closely related genus, *Kermes echinatus* [2,20] and *K. nahalaii* [18,21], *A. galliformis* has a substantially more robust prothorax with a broader connection to the head; the body also tapers more strongly posterior to the scutum, and the genital segment is narrower. Furthermore, the setae of the glandular pouch in *A. galliformis* are thicker and longer; only pleural and ventral setae on the abdominal segments are present as the dorsal abdominal setae are lacking, whereas they are not in *Kermes* species.

Currently, it is not possible to distinguish between *Allokermes* species using routine molecular techniques such as the COI barcode [3,22], and alternative molecular identifications [23] have not been explored. Our description of *A. galliformis* males is therefore a promising new tool for correctly identifying these scales at the species level, as other identification tools remain limited [7,24]. However, finding male specimens, slide mounting, and identifying kermes scales using morphological characteristics can be problematic [2], and the burden of identification is still left to only a few specialist taxonomists. Looking forward, developing more molecular markers would serve as a complementary tool for scale insect identification that would allow identifications to be more broadly accessible.

## 5. Conclusions

This paper describes and illustrates the morphological characteristics of the adult male of *Allokermes galliformis* (Riley, 1881) (Hemiptera: Coccomorpha: Kermesidae) collected from drippy blight diseased trees in Colorado, United States of America. Morphological characteristics such as the subtriangular head, tapered body and genital segment, and the number and arrangement of setae can be used to separate *A. galliformis* from *A. kingii*, as well as relatives in the closely related genus *Kermes*. 

## Figures and Tables

**Table 1:** List of abbreviations used to refer to structures of male *Allokermes galliformis* (Riley, 1881).

aed	aedeagus	med	media	ps	penile sheath
al	alar lobe	mr	marginal ridge	rad	radius
aps	abdominal pleural setae	o	ocellus	scp	scape
al	alar lobe	osc	ocular sclerite	scl	scutellum
asc	additional sclerite	pa	postalar	sclr	scutellar ridge
avs	abdominal ventral setae	pcr_2_	precoxal ridge	sct	scutum
ccx	costal complex of wing veins	pdc	pedicel	ser	subepisternal ridge
cl	claw	phr_2_	mesopostphragma	sp	thoracic spiracles (sp_2–3_)
cx	coxa (cx_1–3_)	plr_2_	mesopleural ridge	st	style
dhs	dorsal head setae	plr_3_	metapleural ridge	stn	sternum (stn_1–3_)
dse	dorsal simple eyes	pn_2_	mesopostnotum	tar	tarsus (tar_1–3_)
eps	episternum (eps_2–3_)	pnwp	posterior notal wing process	tb	tibia (tb_1–3_)
f	furca	pocr	postocular ridge	tdgt	tarsal digitules
fm	femur (fm_1–3_)	por	postoccipital ridge	teg	tegula
fp	furcal pit	prar	prealar ridge	tegs	tegular setae
g	genae	prn	lateral pronotal sclerite	tr	trochanter (tr_1–3_)
glp	glandular pouch	prnr	pronotal ridge	ugdt	ungual digitules
glps	setae of the glandular pouch	procr	preocular ridge	vhs	ventral head setae
gts	setae of genital segment	prsc	prescutum	vse	ventral simple eyes
h	hamulohaltera	prscr	prescutal ridge		

**Table 2:** *Allokermes galliformis* (Riley, 1881) flagellar antennal segment lengths and widths. All measurements are given in micrometers (μm). Ranges are displayed, followed by means in parentheses.

Segments	Scape (scp)	Pedicel (pdc)	III	IV	V	VI	VII	VIII	IX	X
Length range	32–55	46–53	95–107	57–82	32–67	55–65	42–69	40–55	38–53	36–42
Length mean	(41)	(49)	(100)	(64)	(53)	(59)	(59)	(47)	(44)	(39)
Width range	38–44	32–32	19–23	19–23	17–21	19–21	19–23	19–25	11–25	19–21
Width mean	(41)	(32)	(21)	(21)	(20)	(20)	(21)	(22)	(22)	(20)

**Table 3:** *Allokermes galliformis* (Riley, 1881) leg segment lengths and widths. All measurements are given in micrometers (μm). Ranges are displayed, followed by means in parentheses.

Leg	Coxa (cx)	Trochanter (tr)	Femur (fm)	Tibia (tb)	Tarsus (tar)	Claw (cl)	Total
Prothoracic length width	84–88 (85) 44–50 (46)	46–53 (50) 23–29 (26)	160–181 (174) 29–38 (33)	160–181 (170) 17–21 (19)	74–92 (82) 15–19 (17)	17–25 (22)	548–626 (591)
Mesothoracic length width	78–84 (81) 42–46 (43)	50–53 (51) 23–27 (25)	155–185 (170) 29–34 (32)	168–181 (174) 17–21 (20)	69–86 (81) 16–21 (19)	19–25 (22)	540–613 (579)
Metathoracic length width	55–65 (59) 38–44 (41)	50–57 (53) 23–29 (25)	126–147 (137) 29–34 (32)	160–181 (169) 19–25 (22)	74–82 (80) 17–21 (19)	21–25 (24)	485–557 (520)

## References

[1] Kosztarab M. (1996). Scale Insects of Northeastern North America: Identification, Biology, and Distribution.

[2] Spodek M., Ben-Dov Y. (2014). A taxonomic revision of the Kermesidae (Hemiptera: Coccoidea) in Israel, with a description of a new species. Zootaxa.

[3] Gullan P.J., Cook L.G. (2007). Phylogeny and higher classification of the scale insects (Hemiptera: Sternorrhyncha: Coccoidea). Zootaxa.

[4] García Morales M., Denno B.D., Miller D.R., Miller G.L., Ben-Dov Y., Hardy N.B. (2016). ScaleNet: A literature-based model of scale insect biology and systematics. Database.

[5] Gullan P.J., Kosztarab M. (1997). Adaptations in scale insects. Ann. Rev. Entomol..

[6] Sirisena U.G.A.I., Watson G.W., Hemachandra K.S., Wijayagunasekara H.N.P. (2013). A modified technique for the preparation of specimens of Sternorrhyncha for taxonomic studies. Trop. Agric. Res..

[7] Hodgson C.J., Hardy N.B. (2013). The phylogeny of the superfamily Coccoidea (Hemiptera: Sternorrhyncha) based on the morphology of extant and extinct macropterous males. Syst. Entomol..

[8] Riley C.V. (1881). A new species of oak coccid mistaken for a gall. Am. Nat..

[9] Baer R.G., Kosztarab M. (1985). A morphological and systematic study of the first and second instars of the family Kermesidae in the Nearctic region (Homoptera: Coccidea). Va. Polytech. Inst. State Univ. Bull..

[10] Bullington S., Kosztarab M. (1985). Revision of the family Kermesidae (Homoptera) in the Nearctic Region based on adult and third instar females. Va. Polytech. Inst. State Univ. Bull..

[11] Sitz R.A., Cranshaw W.S. (2018). Life history of *Allokermes galliformis* (Hemiptera: Kermesidae) in Colorado. Ann. Entomol. Soc. Am..

[12] Sitz R.A., Zerillo M.M., Snelling J., Caballero J.I., Alexander K., Nash K., Tisserat N.A., Cranshaw W.S., Stewart J.E. (2018). Drippy blight, a disease of red oaks in Colorado produced from the combined effect of the scale insect *Allokermes galliformis* and the bacterium *Lonsdalea quercina* subsp. *quercina*. Arboric. Urban For..

[13] Sitz R.A., Aquino V., Tisserat N.A., Cranshaw W.S., Stewart J.E. (2019). Insects visiting drippy blight diseased red oak trees are contaminated with the pathogenic bacterium *Lonsdalea quercina*. Plant Dis..

[14] Cockerell T.D.A. (1898). XXXVII—New North-American insects. J. Nat. Hist..

[15] Hamon A.B., Lambdin P.L., Kosztarab M. (1976). Life history and morphology of *Kermes kingii* in Virginia. Va. Polytech. Inst. State Univ. Bull..

[16] Afini S.A., Kosztarab M. (1969). Morphological and systematic studies on the adult males of some species of *Lecanodiaspis* (Homoptera: Coccoidea: Lecanodiaspididae). Va. Polytech. Inst. State Univ. Bull..

[17] Afini S.A.M. (1969). Systematic status of the family Conchaspididae, based on the males of *Conchaspis lata* Hempel. (Homoptera: Coccoidea). Va. Polytech. Inst. State Univ. Bull..

[18] Sternlicht M. (1969). *Kermes bytinskii* n. spec. (Coccoidea, Kermesidae) in Israel and observations on its life history. Isr. J. Entomol..

[19] Buschbeck E.K., Hauser M. (2009). The visual system of male scale insects. Naturwissenschaften.

[20] Balachowsky A. (1953). Sur les Kermes Boitard (Hom: Coccoidea) des Chenes du Bassin Oriental de la Méditerranée. Revue de Pathologie Végétale et d’Entomologie Agricole de France.

[21] Bodenheimr F.S. (1931). Zur Kenntnis de paläartkischen Kermes-arten (Rhy. Cocc.). Konowia.

[22] Cook L.G., Gullan P.J., Trueman H.E. (2002). A preliminary phylogeny of the scale insects (Hemiptera: Sternorrhyncha: Coccoidea) based on nuclear small-subunit ribosomal DNA. Mol. Phylogenet. Evol..

[23] Marcus J.M. (2018). Our love-hate relationship with DNA barcodes, the Y2K problem, and the search for next generation barcodes. AIMS Genet..

[24] Miller D.R., Miller G.L. (1993). Description of a new genus of scale insect with a discussion of relationships among families related to the Kermesidae (Homoptera: Coccoidea). Syst. Entomol..

